# Morphological response accompanying size reduction of belemnites during an Early Jurassic hyperthermal event modulated by life history

**DOI:** 10.1038/s41598-021-93850-0

**Published:** 2021-07-14

**Authors:** Paulina S. Nätscher, Guillaume Dera, Carl J. Reddin, Patrícia Rita, Kenneth De Baets

**Affiliations:** 1 Geozentrum Nordbayern, Friedrich-Alexander-Universität Erlangen-Nürnberg, Erlangen, Germany; 2 GET, Université Paul Sabatier, CNRS UMR 5563, IRD, Toulouse, France; 3 Museum für Naturkunde, Leibniz Institute for Evolution and Biodiversity Science, Berlin, Germany; 4 MARE (Marine and Environmental Sciences Centre), 3004-517 Coimbra, Portugal

**Keywords:** Ecology, Evolutionary ecology, Palaeoecology, Palaeontology, Climate-change ecology

## Abstract

One of the most common responses of marine ectotherms to rapid warming is a reduction in body size, but the underlying reasons are unclear. Body size reductions have been documented alongside rapid warming events in the fossil record, such as across the Pliensbachian-Toarcian boundary (PToB) event (~ 183 Mya). As individuals grow, parallel changes in morphology can indicate details of their ecological response to environmental crises, such as changes in resource acquisition, which may anticipate future climate impacts. Here we show that the morphological growth of a marine predator belemnite species (extinct coleoid cephalopods) changed significantly over the PToB warming event. Increasing robustness at different ontogenetic stages likely results from indirect consequences of warming, like resource scarcity or hypercalcification, pointing toward varying ecological tolerances among species. The results of this study stress the importance of taking life history into account as well as phylogeny when studying impacts of environmental stressors on marine organisms.

## Introduction

The most common responses of recent and fossil marine invertebrates to global warming are extinctions^1^, changes in geographical distribution^2^, phenology^3,4^, and decreases in body size^5–10^. However, many biological mechanisms may underlie body size reductions^8,10–13^. Generally, individuals in colder environments grow more slowly but become larger as adults^14^. This widespread pattern is embodied by two well-established hypotheses: Bergmann's rule, which describes the negative association between temperature and body size in natural environments^15^, and the temperature-size rule (TSR)^16^, which describes physiological reaction norms relating temperature to body size in laboratory experiments^8,11,17,18^. These hypotheses predict that organisms should grow to be larger in colder environments, because growth efficiency decreases with increasing environmental temperature^14^. Whether through metabolic processes^19,20^ or reduced resource availability, exposure to higher temperatures and accompanying factors like increased *p*CO_2_ during early development have been shown to lead to altered growth processes^21–23^. Possible outcomes include abnormal growth during the juvenile life stage, or precocious maturity^13,24^, which might lead to paedomorphosis, a heterochronic change marked by retention of juvenile features in adult organisms^5^. While a decrease in body size has been reported from various past hyperthermal events^25–33^, morphological changes of different life stages are not commonly studied^34^ although they are crucial for understanding long-term impacts on life history and their ecological implications.

Here we tackle this issue by focusing on one of the most severe extinction crises of the Jurassic: the Pliensbachian-Toarcian crisis. This event was a global, multi-phased, hyperthermal event triggered by volcanic episodes in the Karoo-Ferrar Large Igneous Province^35–38^, which led to the extinction of around 15–20% of marine species^39^. This disturbance was characterized by an early abrupt warming of at least 5° C at the Pliensbachian-Toarcian boundary event (PToB)^40–43^, which is considered a precursor crisis to the early Toarcian warming and oceanic anoxic event (T-OAE)^44,45^.

**Figure 1:**
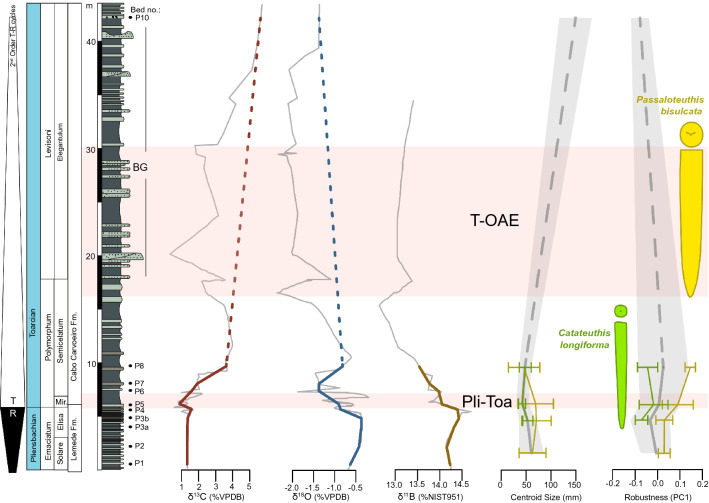
Palaeoenvironmental proxies in the Peniche GSSP section fluctuate across two hyperthermal pulses during the Pliensbachian-Toarcian crisis (PToB and T-OAE). The proxies from left to right are: 2nd order transgressive–regressive cycles to visualise changing bathymetry^87^, δ^13^C as a proxy for carbon cycle disturbances^42^, δ^1^^8^O as palaeotemperature proxy^42^, and δ^11^B as a proxy for ocean acidification^42^. The grey lines in the background of the isotope curves show the original values^42^, whereas the thicker lines overlaying them represent the lower-resolution curves with data points only at the positions of the stratigraphic beds sampled in this study (see stratigraphic log on the left). The second-to-right curves show median GPA Centroid size as a body size proxy (grey line shows full assemblage pattern, with shaded area representing median absolute deviation (MAD), *C. longiforma* and *P. bisulcata* are shown in green and yellow, respectively, with error bars representing MAD). On the right-hand-side PC1 is displayed as proxy for robustness in purple (for colouration see Centroid Size description). The two belemnite shapes bracketing the robustness curves show typical cross section appearances for *C. longiforma* (in green on the left) and *P. bisulcata* (in yellow on the right).

The Peniche GSSP section (Portugal) offers an excellent geochemical and fossil record of the PToB crisis (Fig. 1). Trace fossils are present throughout the section, indicating consistently oxygenated bottom waters^46^. Sedimentological and geochemical proxies show the rapid warming event^40–43^ and sea-level transgression at the PToB^45^ with evidence for ocean acidification^42^, increased weathering^41^, collapsed productivity and resource scarcity^40,47^, which was followed in the early Toarcian by a second, more severe environmental crisis (the T-OAE) (Fig. 1). The temporary disappearance of macroinvertebrates like belemnites (Fig. 1), additionally indicates harsh conditions during the T-OAE^48^. Belemnites are among the organisms that suffered considerably during this crisis^48–52^. Their abundance in the fossil record^48,53–55^, and rapid response to environmental changes, makes these predators suitable for studying responses to past climate changes.

A significant size decrease in adults of the dominant belemnite species was evidenced across the PToB in Peniche^48^, while the extinction of forms with slender belemnite rostra but consistent diversity of robust forms is interpreted as a selective extinction against deep-water dwelling slender forms which were sensitive to decreased oxygen concentrations^51^.

Here we use 3D landmark analysis^56^ to quantify morphological changes of belemnites across the PToB crisis in Peniche. Basing our hypotheses on prior studies, we investigate, whether the size reduction across the boundary was accompanied by allometric changes and thus affected certain life stages disproportionally. Firstly, we expect the largest shift in morphospace (see results and methods) across the PToB as an increase in robustness^48,51^. If this was driven by accelerated growth, we expect a within-species morphological change driven by paedomorphosis of the best represented species^48^. We test for morphological changes on the (1) assemblage and (2) species level and (3) by life stage. Furthermore, we expect these changes to be correlated with increased temperature, rather than with severe shifts in depositional environments.

## Results

### Belemnite morphospace structuration

The consensus shape produced through the Generalized Procrustes Analysis (GPA) is a ventral to dorsal profile through an almost cylindrical belemnite rostrum of medium robustness. The alveolar walls at the reference point on transversal sections are of average thickness and the protoconch is located close to the centre of the rostrum with almost equal distance to the dorsal and ventral sides^57^. The first axis of the Principal Components Analysis (PCA) represents the ratio of width to length (robustness) of the cross-section and accounts for 82% of morphological variation in the data. PC2 explains 7% of the variance and the morphological variation is only nuanced, contrasting thin from thick alveolar walls in the transversal section representing another aspect of rostrum robustness. Based on the percentage of explained variance, we use PC1 as our main morphology proxy, and use ‘PC1’ and ‘robustness’ interchangeably.

**Figure 2:**
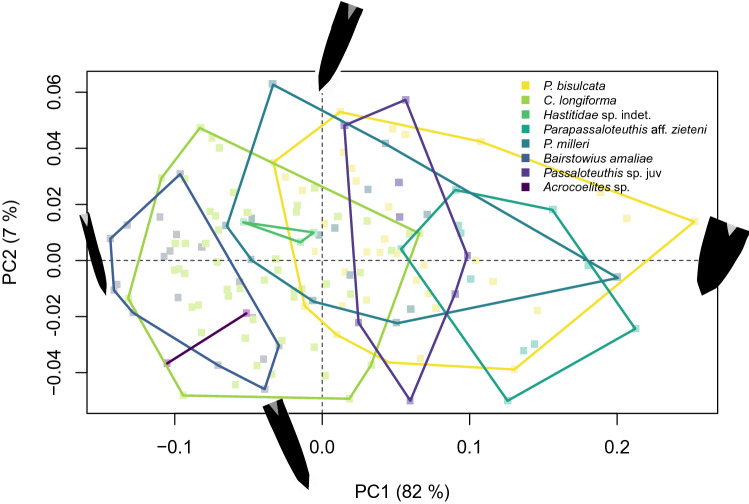
Subspace occupation within the two-dimensional morphospace (PC1, PC2) of the taxa present in this study. Points represent specimens and are coloured by taxon. The convex hulls connect the outermost values on PC1 and PC2 of each individual taxon. The black shapes around the figure represent the maximum and minimum shapes of the sagittal section through the belemnite rostrum of PC1 and PC2. (see sample sizes in Table 1)

The studied species occupy different regions of the morphospace (Fig. 2, supplementary table S1). More slender species include *Bairstowius amaliae*, *Catateuthis longiforma*, *Acrocoelites* sp. and indeterminable Hastitidae specimens. Out of these, the robustness of *C. longiforma* and *B. amaliae* differs significantly (*p* = 0.014). *Passaloteuthis milleri* and *Passaloteuthis bisulcata* show a wide range in terms of robustness from moderately slender to very robust shapes. Significant differences between both of these more robust species and *B. amaliae* are found (*P. milleri*, *p* < 0.001, *P. bisulcata*, *p* < 0.001). Additionally, the robustness of *C. longiforma* is significantly lower than both, *P. milleri* and *P. bisulcata*, too (*P. milleri*, *p* = 0.003, *P. bisulcata*, *p* < 0.001). *Parapassaloteuthis* aff*. zieteni* is, on average, the most robust species, differing significantly from all other species (*P. bisulcata*, *p* = 0.006, *P. milleri*, *p* < 0.001, *B. amaliae*, *p* < 0.001, *C. longiforma*, *p* < 0.001) (supplementary table S1).

### Morphology through time

The full assemblage shows both a small but significant size decrease across the Pliensbachian-Toarcian boundary (Hedges’ g = − 0.022, *p* = 0.018), as well as a large, significant size increase from the *Dactylioceras semicelatum* to the *Elegantuliceras elegantulum* subzone after the T-OAE (Hedges’ g = − 6.302, *p* = 0.006), in which only two specimens of the species *Acrocoelites* sp. appear after a comprehensive loss of belemnite abundance and diversity^48,58^. The body size pattern of the full assemblage is not accompanied by any significant changes in robustness (Fig. 1, supplementary table S3).

The bootstrapping procedure (see methods) shows that the change in species abundances through the subzones is within the realm of random sampling, with the only exception being the *Elegantulum* subzone (supplementary Fig. S2). Because this sample is from after the T-OAE and the changes in community composition across the boundary appear to be random, we reject the hypothesis that community composition markedly influenced the morphological pattern across the PToB.

For species-level analyses, we focus on *P. bisulcata* and *C. longiforma*, which dominate the assemblages across the boundary and are reasonably represented in our morphological subset*. Catateuthis longiforma,* and to a lesser extent *P. bisulcata* have been shown to decrease in body size in response to the PToB event^48^. While the sample of *P. bisulcata* in our dataset does not change in size (supplementary table S2), *C. longiforma* shrinks significantly across the boundary (*p* < 0.001) and increases in body size again in the following subzone (*p* = 0.015) (Fig. 1, supplementary table S2). As assumed (see methods), both species become more robust across the boundary (*P. bisulcata:* Hedge’s g = − 1.054, *p* = 0.043, *C. longiforma*: Hedge’s g = − 0.844, *p* = 0.02) (Fig. 1, supplementary table S3). After this initial increase in robustness, *C. longiforma* returns to a slenderer shape in the *Dactylioceras semicelatum* subzone (Hedges’ g = 0.229), while *P. bisulcata* becomes even more robust (Hedges’ g = − 1.165) (Fig. 1, supplementary table S3). However, these changes from the *Dactylioceras mirabile* subzone to the *Semicelatum* subzone are not significant (supplementary table S3).

**Figure 3:**
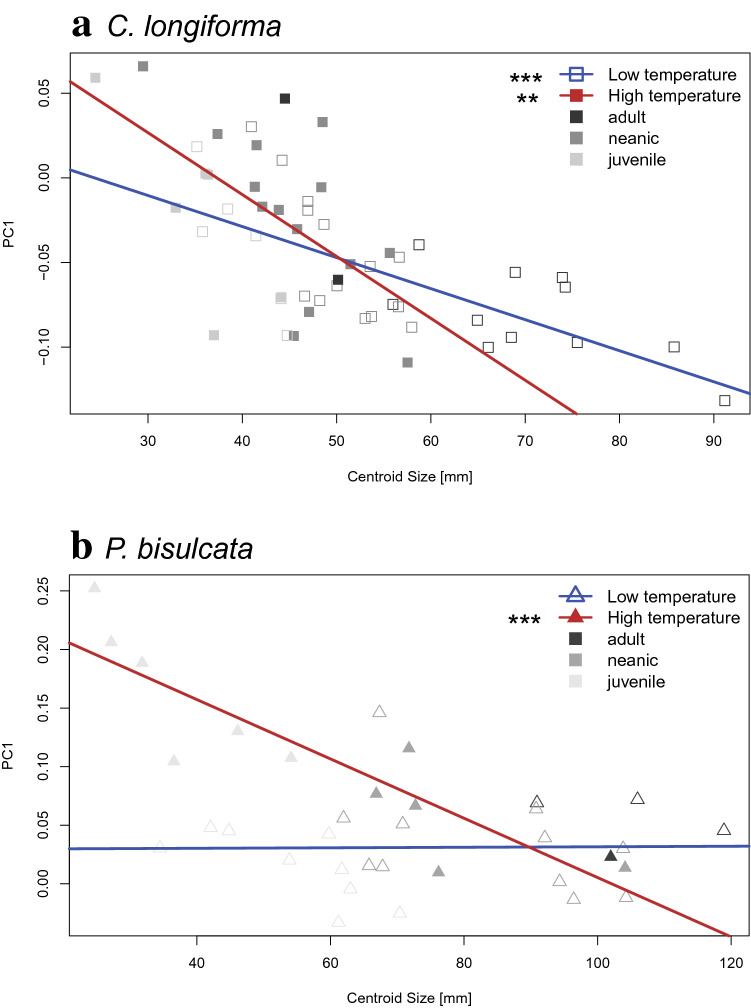
Linear regression lines of GPA Centroid Size with PC1 for cooler (blue line; empty points) and warmer (red line; filled points) times in *C. longiforma* (**a**) and *P. bisulcata* (**b**). The shade of the points indicates ontogenetic stage (light grey: juvenile, medium grey: neanic, black: adult). Significance levels of the regressions are indicated next to the legend.

In both species, smaller specimens are generally more robust (Fig. 3). The slope of robustness at size becomes steeper in higher temperatures in both species (*P. bisulcata* – warm: slope = − 0.003, adj. R^2^ = 0.778, *p* < 0.001, *P. bisulcata*—cold: slope =− 0.001, adj. R^2^ = − 0.047, *p* = 0.954, *C. longiforma –* warm: slope = − 0.004, adj. R^2^ = 0.309, *p* = 0.004, *C. longiforma—*cold: slope = − 0.002, adj. R^2^ = 0.458, *p* < 0.001), meaning that the differences in robustness among life stages are less noticeable in lower temperature settings, while they become more pronounced in high temperature habitats (Fig. 3). The difference in growth trajectories between warm and cold settings is insignificant in *C. longiforma* (*p* = 0.138), but significant for *P. bisulcata* (*p* < 0.001).

**Figure 4:**
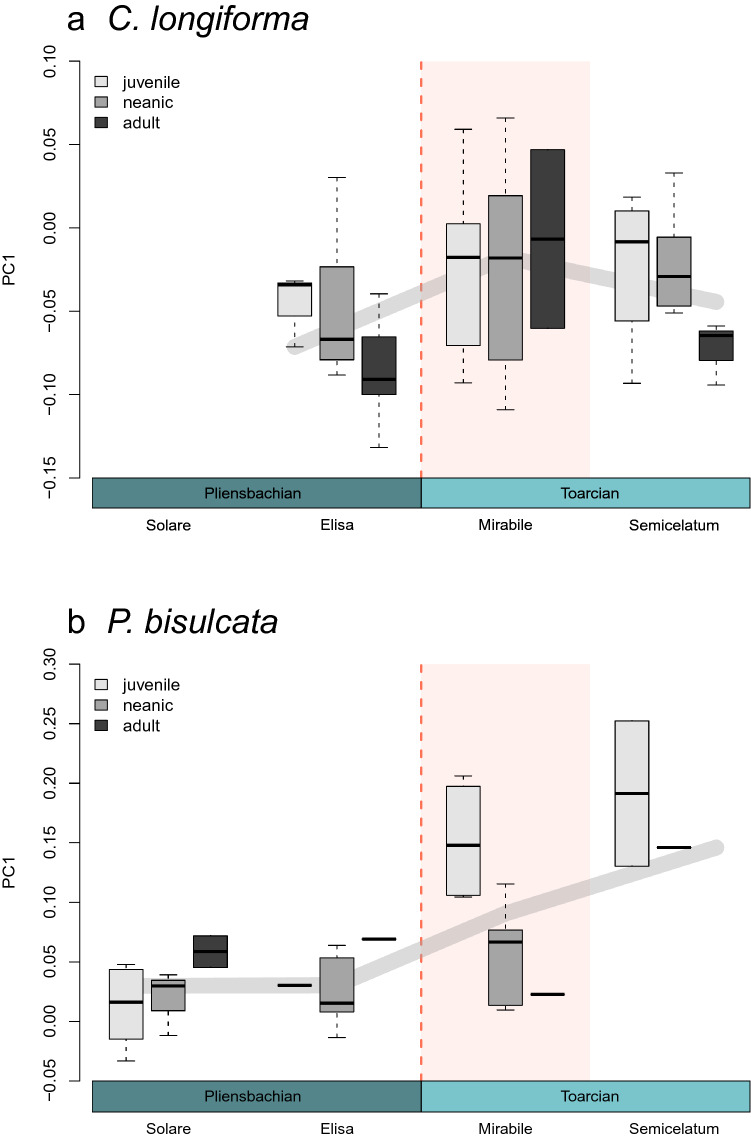
Robustness (PC1) of (**a**) *C. longiforma* and (**b**) *P. bisulcata* life stages by subzone. The thick grey lines indicate the overall robustness, while the overlaying boxplots are split by ontogenetic stage (left to right: juvenile (light grey), neanic (medium grey), adult (dark grey)). The red background in the first Toarcian subzone (Mirabile) indicates the hyperthermal stress from the PToB event. (see sample sizes in Table 1).

The linear regression model produced significant evidence for developmental stage being a good predictor of morphological change in *C. longiforma* (*p* = 0.024) (supplementary table S4). *Catateuthis longiforma* shows a negative allometric growth during both Late Pliensbachian subzones, defined as background times, with older life stages being more slender than juveniles (Fig. 4). The older life stages (neanics and adults) become more robust across the boundary (Hedges’ g = − 0.904, *p* = 0.054) (supplementary table S5), making it look like the growth pattern switches to isometric growth during the hyperthermal event (Fig. 4). In *P. bisulcata* adults basically disappear after the boundary and juveniles become significantly more robust over time (*p* = 0.005) (Fig. 4). However, this seems to be happening gradually, as differences between consecutive subzones are insignificant (supplementary table S5).

The independent effects of temperature reflected by oxygen isotopes (*p* = 0.002), seawater pH, reflected by boron isotopes (*p* = 0.028), and lithology (*p* = 0.001) on overall morphological variation are significant, out of which lithology plays the biggest role with 1.4% (supplementary Fig. S4, supplementary table S6). However, this cannot explain any changes across the boundary as the lithology variable in our samples stays constant there. When correcting for the influence of lithology to identify correlations between robustness and environmental variables, the null model is the most parsimonious for the full assemblage (supplementary table S7). Therefore, no environmental factor seems to consistently influence the overall robustness pattern. For *P. bisulcata* the model including boron isotopes as proxy for acidification is the most parsimonious (supplementary table S7) and performs significantly better than the null model (*p* = 0.014) (supplementary table S8). In *C. longiforma* all variables together, δ^18^O, δ^13^C and δ^11^B, best explain the robustness pattern through time (supplementary table S7). In this model, all environmental variables have a significant correlation with robustness (δ^18^O: *p* = 0.001, δ^13^C: *p* = 0.01, δ^11^B: *p* = 0.002) (supplementary Table S8). These results do not change drastically, when using palaeoenvironmental proxies (δ^18^O, δ^13^C, and Hg/TOC) from different publications^38,43,59^, except for the significant correlation of the morphological pattern of *P. bisulcata* with the acidification proxy (δ^11^B), which is not present in the older geochemical publications (supplementary tables S9 & S10).

## Discussion

Belemnites were likely dominant in epipelagic habitats (0–150 m)^55^ and fast-swimming nektonic predators^60^, and consequently must have had a demanding metabolism^61^. Even though we find no significant change in robustness of the full assemblage across the PToB, the two dominant species (*C. longiforma* and *P. bisulcata*) both show a significant increase in robustness at the event, which can be attributed to direct and indirect consequences of the warming of the environment, like changes in continental run-off, ocean acidification and resource sparsity^40–42,46,62^. At the onset of the T-OAE, a shift towards more robustly shaped taxa (often interpreted as more nektobenthic, shallow water inhabitants) was interpreted as an effect of anoxia in deeper-water habitats, inhabited by more slender, pelagic belemnite taxa^51,55,61,63,64^. However, in our material, the random change in relative species abundances across the boundary would be unlikely to impact the increase in assemblage-scale robustness, which leads us to discard the possibility of bathymetric changes or taphonomic processes having a large impact on the morphological signal. If these processes had been substantial, they would have resulted in preferential preservation of more robust species^65,66^, or an environmentally triggered shift of assemblage composition, which is not the case in our data across the PToB.

We interpret the change in morphology of adult specimens that occurs parallel to the decrease in size in *C. longiforma* as an indication for a paedomorphosis by progenesis, *i.e.* the retention of juvenile morphology in stunted adult specimens^5,34,67–70^. A body size decrease in adults of *C. longiforma* has been observed in the full data set of belemnites in Peniche before^48^. This conclusion is further supported by a correlation of the morphological pattern with environmental changes in palaeotemperature, seawater pH and carbon cycle and the fast recovery after the crisis. In this species juveniles show a lower sensitivity to climate warming than adults. Differential sensitivities of life stages to climate-related stressors (CRS) have been found in many recent metazoan groups^71–74^. It has been argued that particularly very early life stages and spawning adults of marine ectotherms might be particularly susceptible to CRS^8,71,75^, while larger juvenile and young adults should be more resistant. If juvenile growth and reproduction is hindered, this likely will impact their population success^24^, which, if happening over larger parts of their geographic range, augments their extinction risk^76,77^. *Catateuthis longiforma* appeared to respond to the warming event by developmental changes resulting in smaller and more robust adults, and then recovered in morphology and population structure following the initial warming event. This suggests its changes in life history strategy allowed this species to more successfully cope with the initial warming event, which is reminiscent of observations on modern ectotherms^11,13^.

Although the sample sizes of the different *P. bisulcata* ontogenetic stages are too small for sound ecological inferences, juveniles of this species sampled after the PToB appeared to show abnormal robustness patterns. If future morphological studies validated this pattern, this could be related to a change in growth in the early development. Many experimental studies on modern squid show that several climate-related stressors can change the morphology of squid’s internal skeletons^24,78–81^. In particular, decreased pH in seawater is associated with abnormal early development of statoliths^81^ and cuttlebones in decabrachians as well as hypercalcification^23,78–80,82^. All of these factors would affect buoyancy and locomotion, affecting the ability of young decabrachians to thrive as normal^23,24,81^. Furthermore, experiments showed that sepiids reared under low feeding conditions develop wider and shorter (more robust) internal shells^83^, and the PToB event shows evidence for both ocean acidification^42^ and low productivity^62^. Because the robustness variation of *P. bisulcata* correlates significantly with seawater acidification, we suggest that hypercalcification and consequently underfeeding are plausible mechanisms for the morphological changes in *P. bisulcata,* that should be investigated further.

Given that both of the species that exhibit a significant increase in robustness are affected with arguably differing severity, we propose that *P. bisulcata* and *C. longiforma* may have had different thermal and/or bathymetric niches. The thermal tolerance of *C. longiforma* is unknown, but we argue that, because the higher relative abundance of this species at lower latitude localities^84^, and because its robustness recovered when temperature at the site decreased after the boundary event, *C. longiforma* appears better to be able to adapt to warmer habitats than *P. bisulcata*. Generally, southern range margins of high latitudinal species are believed to be set by interspecific competition, while northern limits are set by physical tolerance^85,86^. We interpret the responses of *P. bisulcata* as indicating that this species was adapted to relative cold to temperate environments, which is consistent with their oxygen isotope signals^50,55^. However, some studies interpreted the family Passaloteuthididae (including both *Catateuthis* and *Passaloteuthis*) as ‘all-rounders’ that could easily migrate vertically or spatially, because of the mix of specialised ecomorphological features they have^51^. Geochemical studies on *Passaloteuthis* specimens concluded that they preferentially dwelled in deep water without seasonal influence^50,55^. The epipelagic habitat in Peniche^87^ and the high abundance of this species during the latest Pliensbachian sea-level regressive phase makes this seem unlikely^48^. Additionally, the sea-level rise after the Pliensbachian-Toarcian boundary^87^ likely created accommodation space that should have favoured *P*. *bisulcata*, aiding their recovery.

Our study found promising evidence for differential susceptibilities to CRS across the life stages of an extinct epipelagic predator, and possibly varying CRS susceptibility between species modulated by their thermal preferences. Further research on thermal niches and varying sensitivities among life stages in the fossil record can help anticipate CRS vulnerability in modern organisms^88^ and inform conservation efforts.

## Material and methods

### Materials

930 belemnites were collected during multiple field trips to Peniche (Portugal) in 2016, 2017 and 2018. Specimens were collected from ten consecutive beds in three ammonite zones (*Emaciatum*, *Polymorphum* and *Levisoni*) covering five subzones (*Solare*, *Elisa*, *Mirabile*, *Semicelatum* and *Elegantulum*). Two different sampling techniques were employed to reduce the impact of sampling bias^48^. First, specimens were quantitatively sampled by throwing a 1 m^2^ frame three times per bed and collecting all belemnites and fragments that were found within that frame. Afterwards 30 complete specimens were sampled from each bed to facilitate taxonomic identification. Taxonomy and ontogenetic stages were identified based on traditionally used morphological traits^48,57,84,89,90^. Out of the 930 belemnites^48,84^, all sufficiently preserved specimens were chosen for this study and represent the full samples’ taxonomic composition and ratio of ontogenetic stages well^48^. The resulting dataset consists of 144 belemnites from seven species in three subfamilies (Table 1, supplementary Fig. S1).

**Table 1:** Table of occurrences of species in the five ammonite subzones in the format: absolute abundance (adult|neanic|juvenile).

	*P. bisulcata*	*C. longiforma*	*Bairstowius amaliae*	*P. milleri*	*Parapassaloteuthis* sp*.*	*Passaloteuthis* sp. juv	*Hastitidae* indet	*Acrocoelites* sp.
*Elegantulum*	–	–	–	–	–	–	–	**2**(2|0|0)
*Semicelatum*	**3** (0|1|2)	**13** (3|6|4)	–	**3** (1|0|2)	**4** (3|0|1)	**1** (0|0|1)	–	–
*Mirabile*	**10** (1|5|4)	**17** (2|10|5)	–	–	–	–	**3** (indet.)	–
*Elisa*	**9** (1|7|1)	**23** (8|12|3)	**8** (1|6|1)	**3** (1|2|0)	**7** (3|0|4)	**2** (0|0|2)	–	–
*Solare*	**13** (2|3|8)	–	**10** (2|4|4)	**8** (3|5|0)	–	**5** (0|0|5)	–	–

These belemnites were scanned in the micro-CT scanner (v|Tome|X) of the GeoZentrum Nordbayern. After reconstructing the CT scans using VG Studio max, the open source software 3D Slicer^91^ was used to virtually clean the belemnite rostra from encrusting organisms, adhering sediment and the sediment filling the alveolus. The rostrum cavum was unevenly fractured at different lengths in most specimens. To correct for this taphonomic bias, we cut the rostrum cavum of all 3D models to 1/6th of the length of the rostrum solidum, as this was a length that was preserved in the available specimens. The specimens are stored at the Museu da Ciência da Universidade de Coimbra (Portugal) and the tomographic data can be accessed on MorphoBank (see Data Availability).

### Methods

To attain proxies for palaeoenvironmental changes across the PToB, we used WebPlotDigitizer-4^92^ to extract δ^18^O (temperature proxy), δ^13^C (carbon cycle proxy) and δ^11^B (seawater pH proxy) values from a geochemical study of the same outcrop^42^ and assigned them to the corresponding beds in our data^48^ and consequently all belemnite rostra found within each bed.

**Figure 5:**
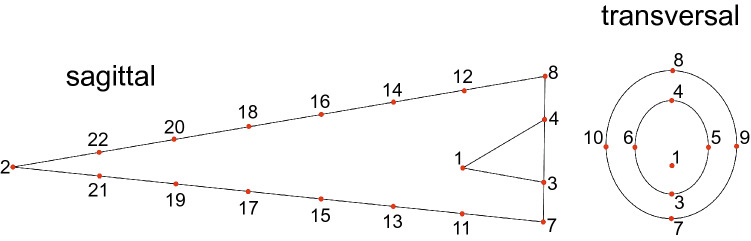
The positions of the 22 landmarks on the sagittal (left figure) and transversal cross section (right figure) of a schematic belemnite rostrum: The rostrum cavum is cut to 1/6th of the length of the rostrum solidum, landmarks 11–22 are curve-sliding semi-landmarks that orientate themselves equidistantly along the ventral and dorsal side of the sagittal plane through the rostrum to counteract potential inaccuracies through human error.

The ‘geomorph' R package^56^ was used to manually place 22 3D-landmarks (Fig. 5) on the belemnite rostra. The landmarks that were placed on the dorsal and ventral sides of the rostra were defined as curve sliders. This means they would orientate themselves equidistantly along the flanks using minimum bending energy and can therefore counteract potential inaccuracies in placing them. A 3D-array, produced with the ‘abind’^93^ package, of the landmarks of all specimens is used for the General Procrustes Analysis (GPA)^56,94^. This method rotates and scales the landmarks of all specimens in 3D space to the point of highest similarity. It then calculates the consensus shape (= mean) of all specimens^95^. The ’Procrustes distances’ between the specimens and the ’consensus shape’ are used to perform a Principal Component Analysis (PCA).

First, we run a linear model on the relationship between the geometric mean and centroid size (supplementary Fig. S3). The former has been used as a body size proxy successfully before^48^, and centroid size is the size component of a specimen that gets removed during the Procrustes transformation in geometric morphometrics^56^. This is done to test, whether we could use centroid size as a reliable estimate of body size in this study.

To test for changes in morphology on different levels of assemblage organisation, we use Hedge’s g from the ‘effsize’^96^ package to quantify changes over time, and assess statistical significance with Mann–Whitney U tests between consecutive samples.

To test, whether a change in species composition of the assemblages has an impact on the morphological pattern, we first use an ANOVA to test for differences in robustness among the species. We subsequently use a bootstrapping method^97^ to test whether changes in species composition across the subzones are random or not. A vector of the species names was drawn from 29 times (mean sample size on subzone level) with replacement, with the relative abundance of each species in the overall sample defined as the probability for it to be selected. This was repeated 500 times. For each of the 500 randomly calculated samples, the simulated relative abundances for the species were calculated. The range of relative abundances of the individual species is defined as the frame, within which changes in abundance can be considered random.

We then test for a within-species change in robustness, specifically across the boundary. The only two species that cross the boundary are *P. bisulcata* and *C. longiforma*. As both these species are known from the full dataset to have a significantly reduced body size after the boundary, we use Mann–Whitney U tests on those species’ centroid sizes between consecutive subzone samples to test whether this works in our subset of specimens. We repeat this to test for changes in robustness (PC1) of these species through time.

To test, whether life stages within the species were affected differently we first employed a linear regression model, using developmental stage as an ordinal predictor variable. The ordinal data structure was used, because it shows trends in smaller data sets more reliably than categorical data structures. We then calculated AICc for both, the null-model and the model containing developmental stage to assess parsimony. Additionally, we run a mood’s median test^98^ on the robustness of the life stages of both dominant species among subzones. Because of lower sample sizes in the adults of both species, we group adult and neanic specimens together and compare them to juveniles. A post-hoc pairwise median test^99^ is used to show significance of changes in morphology between consecutive subzones. For all statistical tests we presume that sample size is sufficient, when p-values return at least marginally significant results (< 0.1), as insufficient sampling usually leads to type II errors and does not suggest false positive results. We include marginal significance, as lower p-values are less likely to be produced with smaller data sets.

The hypothesis, that accelerated growth at higher temperatures causes the change in morphology in adult specimens, is tested by examining the relationship between size and robustness in both species. We fit two linear regressions for each species. One for specimens found in beds during times of lower temperature (δ^18^O > − 0.9) and one for belemnites that lived in a higher temperature environment (δ^18^O < − 0.9) after the boundary. Low temperature and high temperature is defined based on which stratigraphic beds coincide with hyperthermal pulses of the Pliensbachian-Toarcian crisis. The “low temperature” and “high temperature” models are then compared through an ANOVA.

We use variation partitioning from the ‘vegan’ package^100^ to calculate the independent effects that environmental changes and lithology have on the morphospace,. For this we use four variables: δ^18^O, δ^13^C, δ^11^B as environmental variables, and lithology (categorical: marl, limestone, marly limestone).

To assess the impact of δ^18^O, δ^13^C and δ^11^B on the robustness on the assemblage scale as well as for *P. bisulcata* and *C. longiforma* individually, we use generalised least squares (GLS) modelling from the ‘nlme’ package in R^101^, correcting for the impact of lithology. This approach was decided on, because it corrects for auto-correlated residuals in the linear model. The chosen environmental variables were proxies for seawater temperature (δ^18^O), carbon cycle perturbations (δ^13^C) and seawater acidification (δ^11^B). The correlation between the variables is < 0.7^102^ (supplementary Fig. S5). We determine the most parsimonious model using AICc from the ‘nlme’ library in R^101^, which corrects for uneven sample sizes.

All analyses were conducted in R^103^ (we use the R package ‘viridis’^104^ to create figures accessible to colour blind people).

## Supplementary Information

Supplementary Information.

## Data Availability

Uncompiled image stacks and mesh files of all belemnites in this study, including specimen identification numbers, have been deposited in a Morphobank repository (http://morphobank.org/permalink/?P3873). The data and R code used for data analysis have been deposited in a GitHub repository and will be made publicly available upon publication (https://github.com/PauSofN/Belemnite_Morphology_modulated_by_life_stages).
