# Supplementary material for: Morphological response accompanying size reduction of belemnites during an Early Jurassic hyperthermal event modulated by life history
Source: Sci Rep. 2021 Jul 14;11:14480. doi: 10.1038/s41598-021-93850-0 (PMC8280180; doi:10.1038/s41598-021-93850-0)
Supplement: Supplementary file 1 — Supplementary Information. [file 41598_2021_93850_MOESM1_ESM.pdf]

Supplementary material to:

Morphological response accompanying size reduction of belemnites during an Early Jurassic hyperthermal event modulated by life history

Paulina S. Nätscher[1]*, Guillaume Dera[2], Carl J. Reddin[3], Patrícia Rita[4], Kenneth De Baets[1]

[1] Geozentrum Nordbayern, Friedrich-Alexander-Universität Erlangen-Nürnberg, Erlangen, Germany

[2] GET, Université Paul Sabatier, CNRS UMR 5563, IRD, Toulouse, France

[3] Museum für Naturkunde, Leibniz Institute for Evolution and Biodiversity Science, Berlin, Germany

[4] MARE (Marine and Environmental Sciences Centre), 3004-517 Coimbra, Portugal

* Corresponding author

E-Mail address: paulina.naetscher@fau.de (PSN)

Table S1: p-values of differences in robustness between pairs of belemnite species represented in the dataset. Results were obtained by running an ANOVA and a consecutive Tukey post-hoc test.

**ANOVA between species PC1**	**diff**	**lower**	**upper**	**p**	
Bairstowius sp. A-*Acrocoelites* sp.	-0.014	-0.134	0.106	0.999	
bisulcata-Acrocoelites sp.	0.136	0.018	0.253	0.012	*
Hastitidae sp. indet.-*Acrocoelites* sp.	0.054	-0.094	0.201	0.952	
longiformis-Acrocoelites sp.	0.036	-0.08	0.156	0.979	
milleri-Acrocoelites sp.	0.098	-0.024	0.22	0.215	
Parapassaloteuthis aff. *zieteni-Acrocoelites* sp.	0.204	0.08	0.328	< 0.0001	***
Passaloteuthis sp. juv-*Acrocoelites* sp.	0.134	0.007	0.262	0.032	*
bisulcata-Bairstowius sp. A	0.15	0.103	0.196	< 0.0001	***
Hastitidae sp. indet.-*Bairstowius* sp. A	0.068	-0.033	0.168	0.442	
longiformis-Bairstowius sp. A	0.05	0.006	0.094	0.014	*
milleri-Bairstowius sp. A	0.112	0.055	0.17	< 0.0001	***
Parapassaloteuthis aff. *zieteni-Bairstowius* sp. A	0.218	0.156	0.28	< 0.0001	***
Passaloteuthis sp. juv-*Bairstowius* sp. A	0.148	0.08	0.217	< 0.0001	***
Hastitidae sp. indet.-*bisulcata*	-0.082	-0.179	0.015	0.164	
longiformis-bisulcata	-0.099	-0.135	-0.064	< 0.0001	***
milleri-bisulcata	-0.038	-0.089	0.013	0.321	
Parapassaloteuthis aff. *zieteni-bisulcata*	0.069	0.013	0.124	0.006	**
Passaloteuthis sp. juv-*bisulcata*	-0.001	-0.065	0.062	1	
longiformis-Hastitidae sp. indet.	-0.017	-0.113	0.078	0.999	
milleri-Hastitidae sp. indet.	0.045	-0.058	0.147	0.884	
Parapassaloteuthis aff. *zieteni-Hastitidae* sp. indet.	0.151	0.045	0.256	0.001	***
Passaloteuthis sp. juv-*Hastitidae* sp. indet.	0.081	-0.029	0.19	0.315	
milleri-longiformis	0.062	0.013	0.11	0.003	**
Parapassaloteuthis aff. *zieteni-longiformis*	0.168	0.114	0.221	< 0.0001	***
Passaloteuthis sp. juv-*longiformis*	0.098	0.037	0.159	< 0.0001	***
Parapassaloteuthis aff. *zieteni-milleri*	0.106	0.041	0.171	< 0.0001	***
Passaloteuthis sp. juv-*milleri*	0.036	-0.035	0.108	0.774	
Passaloteuthis sp. juv-*Parapassaloteuthis* sp. 1	-0.07	-0.145	0.005	0.088	

Table S2: Changes in GPA centroid size in *P. bisulcata*, *C. longiforma* and the full assemblage between consecutive ammonite subzones. Effect size is represented by Hedge's g and differences were tested for with Mann-Whitney U tests.

P.bisulcata	**p**	**W**	**Hedges' g**	**lower 95CI**	**upper 95CI**	**Sign. level**
Sol.-Elis.	0.845	55	0.029 (negligible)	-0.841	0.899	
Elis.-Mir.	0.604	52	0.415 (small)	-0.521	1.351	
Mir.-Semic.	0.287	22	0.652 (medium)	-0.721	2.025	
C.longiforma						
Elis.-Mir.	< 0.001	326	1.226 (large)	0.536	1.917	***
Mir.-Semic.	0.015	53	-1.02 (large)	-1.799	-0.24	*
**assemblage**						
Sol.-Elis..	0.146	1108	0.368 (small)	-0.062	0.799	
Elis.-Mir.	0.018	1026	0.435 (small)	-0.022	0.892	*
Mir.-Semic.	0.337	304	-0.015 (negligible)	-0.556	0.527	
Semic.-Eleg.	0.006	0	-6.302 (large)	-8.586	-4.018	***

Table S3: Changes in robustness (PC1) in *P. bisulcata*, *C. longiforma* and the full assemblage between consecutive ammonite subzones. Effect size is represented by Hedge's g and differences were tested for with Mann-Whitney U tests.

P.bisulcata	**p**	**W**	**Hedges' g**	**lower 95CI**	**upper 95CI**	**Sign. level**
Sol.-Elis.	0.431	46	-0.329 (small)	-1.205	0.547	
Elis.-Mir.	0.043	20	-1.054 (large)	-2.042	-0.066	*
Mir.-Semic.	0.077	4	-1.165 (large)	-2.592	0.262	.
C.longiforma						
Elis.-Mir.	0.02	111	-0.845 (large)	-1.506	-0.183	*
Mir.-Semic.	0.592	124	0.229 (small)	-0.508	0.966	
**assemblage**						
Sol.-Elis.	0.943	945	-0.078 (negligible)	-0.506	0.349	
Elis.-Mir.	0.107	612	-0.335 (small)	-0.79	0.12	
Mir.-Semic.	0.402	311	-0.283 (small)	-0.827	0.261	
Semic.-Eleg.	0.055	44	1.218 (large)	-0.291	2.728	.

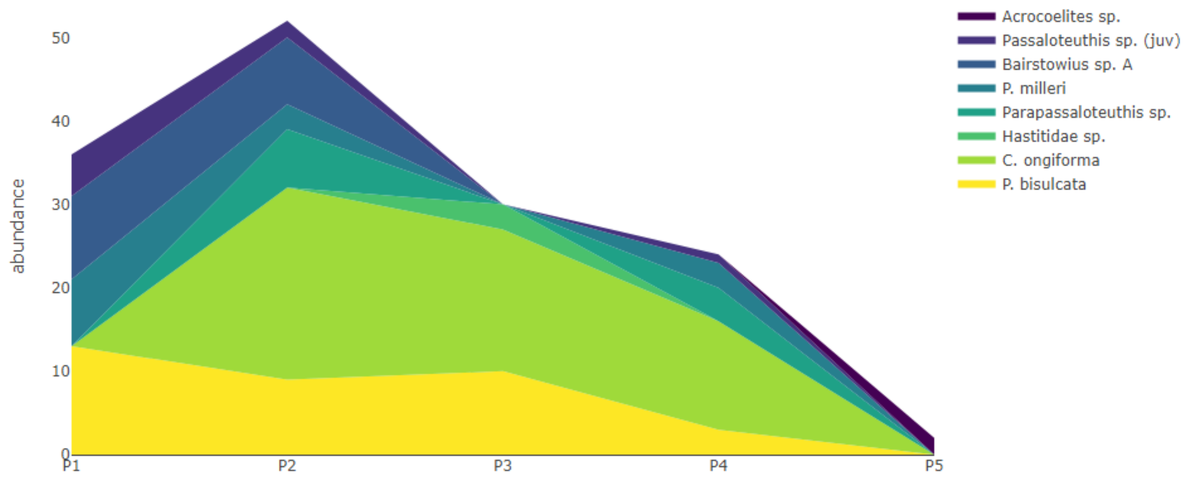

Figure S1: Absolute abundances of the taxa found in this study through the 5 subzones labelled "P1, P2, P3, P4, P5" here, corresponding to Solare, Elisa, Mirabile, Semicelatum, Elegantulum subzones in the manuscript

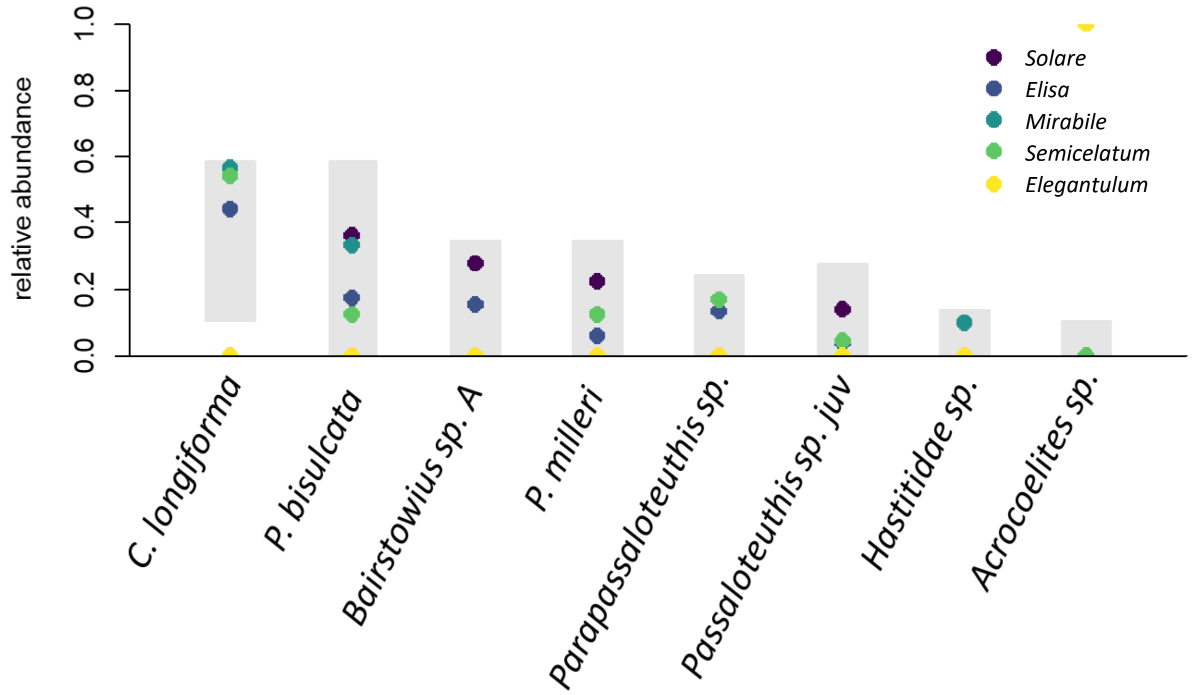

Figure S2: Results of the resampling method to determine non-randomness in changes of community composition over time. Grey rectangles represent the range within the relative abundance of a species in a specific bed is explainable by random sampling, n=29, 500 iterations

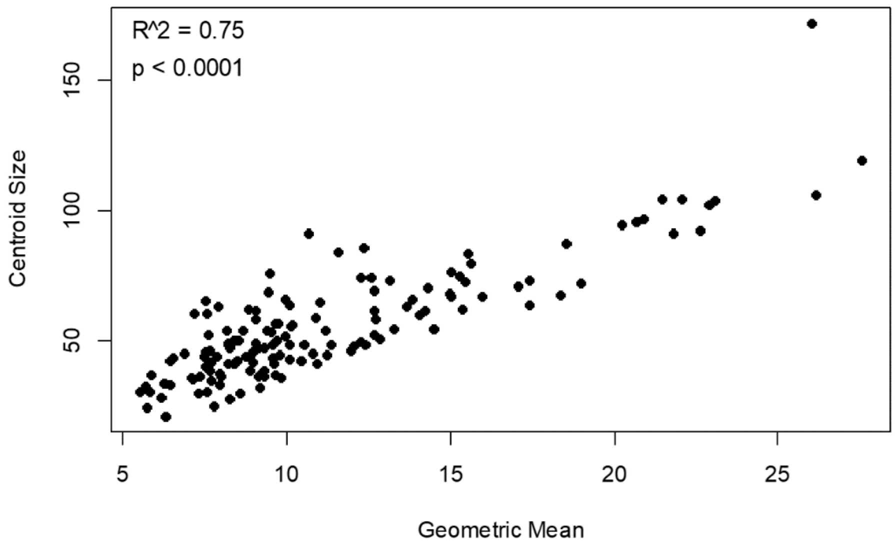

Figure S3: The geometric mean, which has been successfully used as a proxy for body size in previous studies, is highly correlated to centroid size, the size part in geometric morphometrics, which is used for scaling ($R^2 = 0.75$, $p < 0.0001$). Therefore, centroid size is used as a geometric morphometric body size proxy in this study.

Table S4: Linear regression models with developmental stage as an ordinal variable show that life stages do not play a significant role in the morphological variability in *P. bisulcata*. However, in *C. longiforma* developmental stage is a good predictor for morphological variation. Additionally, the linear model that includes life stage shows a more parsimonious fit than the null-model.

P. bisulcata	adj. R2	p	DF	AICc
with dev_stage	0.21	0.202	4	0.751
no dev_stage		0.493		-6.495
C. longiforma				
with dev_stage	0.078	0.024	51	-173.386
no dev_stage		< 0.001		-170.319

Table S5: The results of a Mood's median and post-hoc pairwise median test on the PC1 of both species (*C. longiforma* and *P. bisulcata*) among subzones, show a significant change in robustness in the pooled neanic and adult group across the boundary in *C. longiforma*. Juveniles in *P. bisulcata* change significantly through time, but changes across consecutive subzones are not significant.

	p (Mood's)	X-squared	p (pairwiseMEDIAN adj.)	Hedges' g
C.longiforma				
juveniles	0.123	4.2		
Elis.-Mir.			0.742	-0.374 (small)
Mir.-Semic.			0.777	-0.017 (negligible)
neanic&adult	0.064 **.**	5.512		
Elis.-Mir.			0.054 **.**	-0.904 (large)
Mir.-Semic.			0.268	0.337 (small)
P.bisulcata				
juveniles	0.005 **	10.5		
Sol.-Elis.			0.527	NA
Elis.-Mir.			0.575	NA
Mir.-Semic.			1	-0.502 (medium)
neanic&adult	0.929	0.148		
Sol.-Elis.			0.902	0.079 (negligible)
Elis.-Mir.			1	-0.478 (small)
Mir.-Semic.			0.635	NA

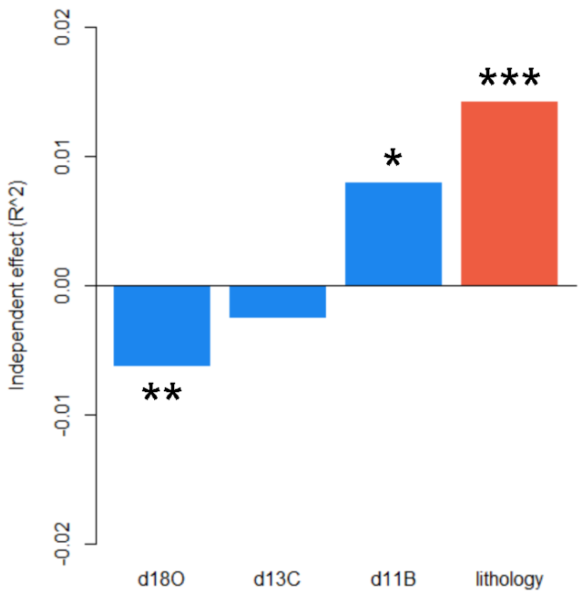

Figure S4: Independent effects of selected environmental and sedimentological parameters on the morphological variance among belemnite rostra. Significance indicated by asterixis (See Table S6).

Table S6: Independent effects of $\delta^{18}O$, $\delta^{13}C$, $\delta^{11}B$ and lithology on the morphological variance of the assemblage, quantified through variation partitioning. The adjusted R^2 and p values were attained through a redundancy analysis.

	**indep. effect**	**rda adj R^2**	**p**	
**$\delta^{18}O$**	-0.006	0.05	0.005	**
**$\delta^{13}C$**	-0.003	-0.003	0.484	
**$\delta^{11}B$**	0.008	0.025	0.028	*
**lithology**	0.014	0.086	0.002	**

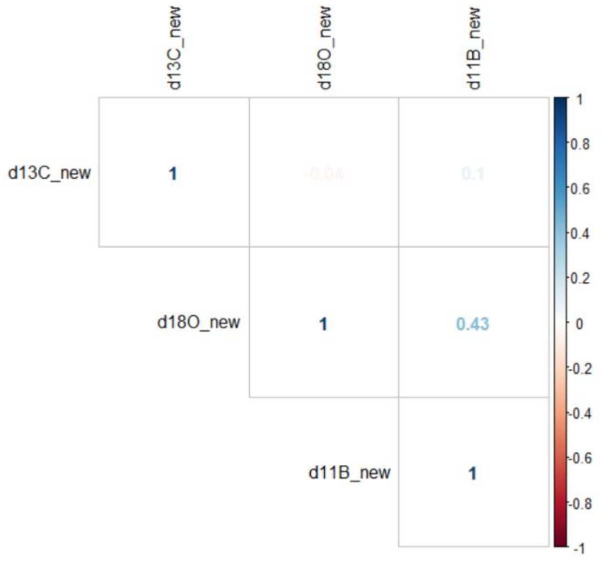

Figure S5: Plot of the correlation coefficients between the environmental parameters used in the gls models ($\delta^{18}O$, $\delta^{13}C$, and $\delta^{11}B$ extracted from Müller et al., 2020).

Table S7: AICc values of the different generalised least squares (gls) models describing the effects of palaeotemperature ($\delta^{18}O$), carbon cycle perturbations ($\delta^{13}C$) and seawater acidification ($\delta^{11}B$) (all from Müller et al. 2020), on the belemnite robustness of the whole assemblage, *P. bisulcata* and *C. longiforma*, corrected for the effect of lithology. The lowest AICc score indicating the most parsimonious model for each of the groups is marked in yellow. For the entire assemblage the null-model is the most parsimonious. Morphological variations in *P. bisulcata* correlate best with boron isotopes as seawater pH proxy, while the full model with all environmental parameters ($\delta^{18}O$, $\delta^{13}C$ and $\delta^{11}B$) is the best model for *C. longiforma.*

**assemblage**	**AICc**
~ 1	**-348.398**
~ $\delta^{13}C$	-346.706
~ $\delta^{18}O$	-346.362
~ $\delta^{11}B$	-341.428
~ $\delta^{13}C + \delta^{18}O$	-344.781
~ $\delta^{13}C + \delta^{11}B$	-339.84
~ $\delta^{18}O + \delta^{11}B$	-339.366
~ $\delta^{18}O + \delta^{13}C + \delta^{11}B$	-337.693
P. bisulcata	
~ 1	-107.336
~ $\delta^{13}C$	-105.537
~ $\delta^{18}O$	-105.109
~ $\delta^{11}B$	**-110.882**
~ $\delta^{13}C + \delta^{18}O$	-103.288
~ $\delta^{13}C + \delta^{11}B$	-108.168
~ $\delta^{18}O + \delta^{11}B$	-109.09
~ $\delta^{18}O + \delta^{13}C + \delta^{11}B$	-106.168
C. longiforma	
~ 1	-168.785
~ $\delta^{13}C$	-166.516
~ $\delta^{18}O$	-170.434
~ $\delta^{11}B$	-167.615
~ $\delta^{13}C + \delta^{18}O$	-168.315
~ $\delta^{13}C + \delta^{11}B$	-166.885
~ $\delta^{18}O + \delta^{11}B$	-170.49
~ $\delta^{18}O + \delta^{13}C + \delta^{11}B$	**-174.333**

Table S8: Statistical parameters of the selected gls models comparing rostrum robustness with palaeotemperature ($\delta^{18}O$), carbon cycle perturbations ($\delta^{13}C$) and seawater acidification ($\delta^{11}B$) (all from Müller et al. 2020), corrected for the effects of lithology. (SE = standard error, DF = degrees of freedom)

**assemblage**	value	SE	t-value	p-value	DF	residual	p (ANOVA best model vs null-model)
Residuals ~ 1 (intercept)	-0.0003	0.001	-0.034	-0.034	144	143	-
P. bisulcata					35	33	0.014
intercept	2.329	0.742	3.137	0.004			
Residuals ~ $\delta^{11}B$	-0.16	0.052	-3.055	0.004			
C. longiforma					53	49	0.005
intercept	1.421	0.435	3.263	0.002			
Residuals ~ $\delta^{18}O$	0.116	0.034	3.428	0.001			
Residuals ~ $\delta^{13}C$	-0.026	0.01	-2.655	0.011			
Residuals ~ $\delta^{11}B$	-0.093	0.029	-3.195	0.002			

Table S9: AICc values of the different generalised least squares (gls) models describing the effects of palaeotemperature ($\delta^{18}O$) (Suan et al. 2008), carbon cycle perturbations ($\delta^{13}C$) (Hesselbo et al., 2007) and volcanism (Hg/TOC) (Percival et al., 2015), on the belemnite robustness of the whole assemblage, *P. bisulcata* and *C. longiforma*, corrected for the effect of lithology. The lowest AICc score, and therefore most parsimonious model, for each of the groups is marked in yellow. For the entire assemblage and *P. bisulcata*, the null-model is the most parsimonious, while the full model with all environmental parameters ($\delta^{18}O$, $\delta^{13}C$ and Hg/TOC) is the best model for *C. longiforma.*

**assemblage**	**AICc**
~ 1	**-348.398**
~ $\delta^{13}C$	-346.765
~ $\delta^{18}O$	-346.29
~ Hg/TOC	-276.394
~ $\delta^{13}C$ + $\delta^{18}O$	-344.625
~ $\delta^{13}C$ + Hg/TOC	-274.214
~ $\delta^{18}O$ + Hg/TOC	-274.23
~ $\delta^{18}O$ + $\delta^{13}C$ + Hg/TOC	-272.014
P. bisulcata	
~ 1	**-107.336**
~ $\delta^{13}C$	-105.839
~ $\delta^{18}O$	-107.046
~ Hg.TOC	-81.645
~ $\delta^{13}C$ + $\delta^{18}O$	-104.32
~ $\delta^{13}C$ + Hg/TOC	-79.156
~ $\delta^{18}O$ + Hg/TOC	-79.619
~ $\delta^{18}O$ + $\delta^{13}C$ + Hg/TOC	-76.373
C. longiforma	
~ 1	-168.785
~ $\delta^{13}C$	-166.725
~ $\delta^{18}O$	-166.97
~ Hg.TOC	-170.407
~ $\delta^{13}C$ + $\delta^{18}O$	-165.113
~ $\delta^{13}C$ + Hg/TOC	-167.976
~ $\delta^{18}O$ + Hg/TOC	-169.551
~ $\delta^{18}O$ + $\delta^{13}C$ + Hg/TOC	**-173.74**

Table S10: Statistical parameters of the selected gls models comparing rostrum robustness with palaeotemperature ($\delta^{18}O$) (Suan et al. 2008), carbon cycle perturbations ($\delta^{13}C$) (Hesselbo et al. 2007) and volcanism (Hg/TOC) (Percival et al. 2015), corrected for the effects of lithology. (SE = standard error, DF = degrees of freedom)

**assemblage**	value	SE	t-value	p-value	DF	residual	p (ANOVA best model vs null-model)
Residuals ~ 1 (intercept)	-0.0003	0.001	-0.034	0.973	144	143	-
**P. bisulcata**							
Residuals ~ 1 (intercept)	0.062	0.018	3.529	0.001	35	34	-
**C. longiforma**							
intercept	0.114	0.062	1.834	0.073	53	49	0.007
Residuals ~ $\delta^{18}O$	0.255	0.084	3.033	0.004			
Residuals ~ $\delta^{13}C$	0.05	0.018	2.724	0.009			
Residuals ~ Hg/TOC	0.365	0.101	3.626	0.001			
